# Supplementary material for: Identification of Potential Druggable Targets and Structure-Based Virtual Screening for Drug-like Molecules against the Shrimp Pathogen Enterocytozoon hepatopenaei
Source: Int J Mol Sci. 2023 Jan 11;24(2):1412. doi: 10.3390/ijms24021412 (PMC9867128; doi:10.3390/ijms24021412)
Supplement: Supplementary file 1 [file ijms-24-01412-s001.zip › Type of the Paper.pdf]

Figure S1. Sequence- and structure-based analyses of the AQP protein of EHP. (**A**) Secondary structure composition of the AQP protein of EHP. The α-helices are labeled as H while the β-strands are represented by purple arrows. (**B**) Multiple sequence alignment of the AQP proteinsof EHP, *A. thaliana*, and human. The global consensus sequence is provided below the alignment. Sequence conservedness is represented by the red bar below the alignment. (**C**)The three-dimensional structure of the AQP protein of EHP generated by AlphaFold2. The different colors in the model representthe model confidence score. (**D**) The quality of the prediction was visualized by the MSA depth and diversity and the confidence measures of AlphaFold2 are provided. (**E**) Predicted local distances between amino acid residues. The inter Predicted Aligned Error (PAE) between chains is very low, indicating that the confidence of the prediction was high. (**F**) Validation of the modeled structures of EHP AQP based on the ProSAZ-score. The Z-scores of all the proteins in the PDB determined by X-ray crystallography and NMR are represented by light blue and dark blue dots, respectively. The Z-scores of the AQP protein of EHP is represented by a large black dot. (**G**) Ramachandran plot of the modeled AQP protein of EHP. The red, yellow, and pale yellow regions within the plot represent the favored, allowed, and generously allowed regions, respectively.

Figure S2. Sequence- and structure-based analysis of the CTP synthase protein of EHP. (**A**) Secondary structure composition of the CTP synthase protein of EHP. The α-helices are labeled as H while the β-strands are represented by purple arrows. (**B**) Multiple sequence alignment of the CTP synthase protein of EHP,drosophila, and human. The global consensus sequence is provided below the alignment. The sequence conservedness is represented as a red bar below the alignment. (**C**) The three-dimensional structure of the CTP synthase protein of EHP generated by AlphaFold2. The different colors in the model represent the model confidence score. (**D**) The quality of the prediction was visualized by MSA depth and diversity and the AlphaFold2 confidence measures are depicted. (**E**) Predicted local distances between amino acid residues. The inter PAE between the chains is very low, indicating that the confidence of the prediction was high. (**F**) Validation of the modeled structure of the CTP synthase protein of EHP based on the ProSAZ-score. The Z-scores of all the proteins in the PDB determined by X-ray crystallography and are represented by light blue and dark blue dots, respectively. The Z-scores of the CTP synthase protein of EHP is represented as a large black dot.(**G**) Ramachandran plot of the modeled CTP synthase protein of EHP. The red, yellow, and pale yellow regions in the plot represent the favored, allowed, and generously allowed regions, respectively.

Figure S3. Sequence- and structure-based analysis of the TK protein of EHP. (**A**) Secondary structure composition of the TK protein of EHP. The α-helices are labeled as H while the β-strands are represented by purple arrows. (**B**) The three-dimensional structure of the TK protein of EHP generated by AlphaFold2. The different colors in the model representthe model confidence scores. (**C**) The prediction quality was visualized by MSA depth and diversity and the AlphaFold2 confidence measures are depicted. (**D**) Predicted local distances between amino the acid residues. The inter PAE between the chains is very low, indicating that the confidence of the prediction was high. (**E**)Validation of the modeled structure of the TK protein of EHP based on the ProSAZ-score. The Z-scores of all the proteins in the PDB determined by X-ray crystallography and NMR are represented by light blue and dark blue dots, respectively. The Z-scores of the TK protein of EHP is represented as a large black dot. (**F**) Ramachandran plot of the model of EHP TK. The red, yellow, and pale yellowregions in the plot represent the favored, allowed, and generously allowed regions, respectively.

Figure S4. Sequence and structure-based analysis of the DHFR protein of EHP. (**A**) Secondary structure composition of the DHFR protein of EHP. The α-

helices are labeled as H while the β-strands are represented as purple arrows. (**B**) Multiple sequence alignment of the DHFR protein of EHP, humans, and *Coxiellaburnetii*. The global consensus sequence is provided below the alignment. The sequence conservedness is represented as a red bar below the alignment. (**C**) The three-dimensional structure of the DHFR protein of EHP generated by AlphaFold2. The different colors in the model representthe model confidence score. (**D**) The prediction quality was visualized by MSA depth and diversity and the AlphaFold2 confidence measures are depicted. (**E**) Predicted local distances between amino acid residues. The inter PAE between the chains is very low, indicating that the confidence of the prediction was high. (**F**) Ramachandran plot of the modeled DHFR protein of EHP. The red, yellow, and pale yellow regions in the plot represent the favored, allowed,and generously allowed regions, respectively. (**G**)Validation of the modeled structure of the DHFR protein of EHP based on the ProSAZ-score. The Z-scores of all the proteins in the PDB determined by X-ray crystallography and NMR are represented by light blue and dark blue dots, respectively. The Z-score of the DHFR protein of EHP is represented as a large black dot.

Figure S5. Sequence- and structure-based analyses of the MetAP2 protein of EHP. (**A**) Secondary structure composition of the MetAP2 protein of EHP. The α-helices are labeled as H while the β-strands are represented by purple arrows. (**B**) Multiple sequence alignment of the MetAP2 proteinsof EHP, *E. cuniculi*, and human. The global consensus sequence is provided below the alignment. The sequence conservedness is represented as a red bar below the alignment. (**C**) The three-dimensional structure of the MetAP2 protein of EHP generated by AlphaFold2. The different colors in the model represent the model confidence scores. (**D**) The quality of the prediction was visualized by MSA depth and diversity and depict the AlphaFold2 confidence measures. (**E**) Predicted local distances between amino acid residues. The inter PAE between the chains is very low, indicating that the confidence of the prediction was high. (**F**) Ramachandran plot of the modeled MetAP2 protein of EHP. The red, yellow, and pale yellow regions in the plot represent the favored, allowed, and generously allowed regions, respectively. (**G**)Validation of the modeled structure of the MetAP2 protein of EHP based on the ProSAZ-score. The Z-scores of all the proteins in the PDB determined by X-ray crystallography and NMR are represented as light blue and dark blue dots, respectively. The Z-scores of the MetAP2 protein of EHP is represented as a large black dot.

Figure S6. Analyses of the MD simulation trajectories of AQP complexed with different compounds, CHEMBL3703838, ZINC000002243083, CHEMBL133039, CHEMBL3140193, CHEMBL3140193, and CHEMBL2132563.(**A–E**) Distribution of the interaction energies of the five protein–ligand complexes. The blue, red, and green lines indicate the interaction energy of the complexes, van der Waals energy, and electrostatic energy, respectively.(**F–J**) 2D diagrams depicting the protein–ligand interactions of the five compounds complexed with the AQP protein after 100 ns MD simulations, determined by LigPlot. The green, purple, blue, and cyan spheres represent aliphatic, aromatic, basic, and polar amino acids, respectively. The green, blue, and purple dotted lines represent hydrophobic interactions, hydrogen bonds, and π-π interactions, respectively.(**K–O**) The dynamical residue cross-correlation matrix of the five compounds complexed with AQP. Values of 1 (pink) and -1 (green) indicate correlated and anti-correlated motions, respectively; the white regions indicate non-correlation.(**P–T**) PCA of the simulation trajectories of the five compounds complexed with AQP. PCA of the resulting trajectory frames changed from white to green to dark green during the simulation.

Figure S7. Analyses of the MD simulation trajectories of the CTP synthase protein of EHP complexed with the five compounds, CHEMBL48494, CHEMBL1162979, CHEMBL133039, CHEMBL1091856, and CHEMBL525202. (**A–E**) Distribution of the interaction energies of the five protein–ligand complexes. The blue, red, and green lines indicate the interaction energy

between the complexes, van der Waals energy, and electrostatic energy, respectively.(**F**–**J**) 2D diagrams of the protein–ligand interactions of the five compounds complexed with CTP synthase after 100ns of MD simulations, determined with LigPlot. The green, purple, blue, cyan, and red spheres represent the aliphatic, aromatic, basic, polar, and acidic amino acids, respectively. The green, blue, and purple dotted lines represent hydrophobic interactions, hydrogen bonds, and π-π interactions, respectively.(**K**–**O**) The dynamical residue cross-correlation matrix of the five compounds complexed with the CTP synthase of EHP. Values of 1 (pink) and -1 (green) indicate correlated and anti-correlated motions, respectively; the white regions indicate non-correlation.(**P**–**T**) PCA of the trajectories of the five compounds complexed with CTP synthase. PCA of the resulting trajectory frames changed from white to green to dark green during the simulation.

Figure S8. Analysis of the MD simulation trajectory of the TK protein of EHP complexed with the five compounds, CHEMBL3674540, CHEMBL1683320, CHEMBL391279, ZINC000031750813, and CHEMBL4078273.(**A**–**E**) Distribution of the interaction energies of the five protein–ligand complexes. The blue, red, and green lines represent the interaction energy between the complexes, van der Waals energy, and electrostatic energy, respectively.(**F**–**J**) The 2D diagrams of the protein–ligand interactions of the five compounds complexed with the TK protein of EHP after 100ns MD simulations, determined with LigPlot. The green, purple, blue, cyan, red, and yellow spheres represent the aliphatic, aromatic, basic, polar, acidic, and sulfur containing amino acids, respectively. The green, blue, and purple dotted line represent hydrophobic interactions, hydrogen bonds, and π-π interactions, respectively. (**K**–**O**) The dynamical residue cross-correlation matrix of the five compounds complexed with TK. Values of 1 (pink) and -1 (green) indicate correlated and anti-correlated motions, respectively; the white regions indicate non-correlation.(**P**–**T**) PCA of the trajectories of the five different compounds complexed with TK. PCA of the resulting trajectory frames changed from white to green to dark green during MD simulations.

Figure S9. Analysis of the MD simulation trajectories of DHFR complexed with the five compounds, CHEMBL1966988, CHEMBL340488, ZINC000016682862, ZINC000828645375, and CHEMBL3901573.(**A**–**E**) Distribution of the interaction energies of the five protein–ligand complexes. The blue, red, and green lines depict the interaction energy of the complexes, van der Waals energy, and electrostatic energy, respectively.(**F**–**J**) 2D diagrams of the protein–ligand interactions of the five compounds complexed with DHFR after 100ns MD simulations, determined with LigPlot. The green, purple, blue, cyan, red, and yellow spheres represent aliphatic, aromatic, basic, polar, acidic, and sulfur containing amino acids, respectively. The green, blue, and purple dotted lines represent hydrophobic interactions, hydrogen bonds, and π-π interactions, respectively. (**K**–**O**) The dynamical residue cross-correlation matrix of the five different compounds complexed with DHFR. Values of 1 (pink) and -1 (green) represent correlated and anti-correlated motions, respectively; the white regions indicate non-correlation.(**P**–**T**) PCA of the trajectories of the five compounds complexed with DHFR. PCA of the resulting trajectory frames changed from white to green to dark green during MD simulations.

Figure S10. Analysis of the MD simulation trajectories of the MetAP2 protein complexed with the five compounds, CHEMBL3913373, CHEMBL1962731, CHEMBL3142997, ZINC000199197855, and ZINC000016682972.(**A**–**E**) Distribution of the interaction energies of the five protein–ligand complexes. The blue, red, and green lines depict the interaction energy of the complexes, the van der Waals energy, and electrostatic energy, respectively.(**F**–**J**) 2D diagrams of the protein–ligand interactions of the five compounds complexed with MetAP2 after 100ns MD simulation, determined with LigPlot. The green, purple, blue, cyan, red, and yellow spheres represent aliphatic, aromatic, basic, polar, acidic, and sulfur containing amino acids, respectively. The green, blue, and purple dotted lines represent hydrophobic interactions, hydrogen bonds, and π-π interactions, respectively. (**K**–**O**) The dynamical residue cross-

correlation matrix of the five compounds complexed with MetAP2. Values of 1 (pink) and -1 (green) represent correlated and anti-correlated motions, respectively; the white regions indicate non-correlation. (P–**T**) PCA of the trajectories of the five compounds complexed with MetAP2. PCA of the resulting trajectory frames changed from white to green to dark green during the MD simulation.
