# Supplementary figures and images for: Identification of Potential Druggable Targets and Structure-Based Virtual Screening for Drug-like Molecules against the Shrimp Pathogen Enterocytozoon hepatopenaei
Source: Int J Mol Sci. 2023 Jan 11;24(2):1412. doi: 10.3390/ijms24021412 (PMC9867128; doi:10.3390/ijms24021412)

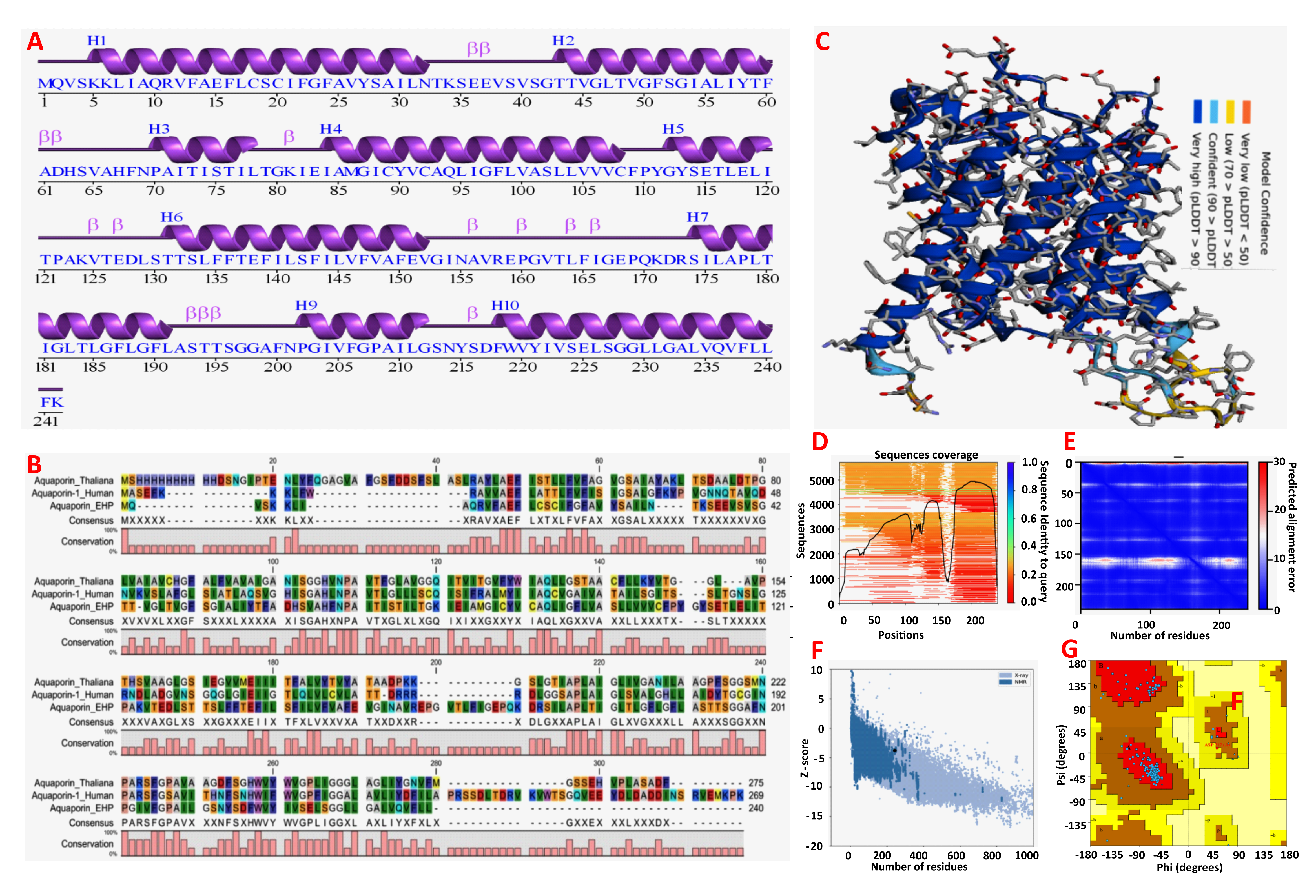

Supplement: Supplementary file 1 [file ijms-24-01412-s001.zip › Figure S1.png]

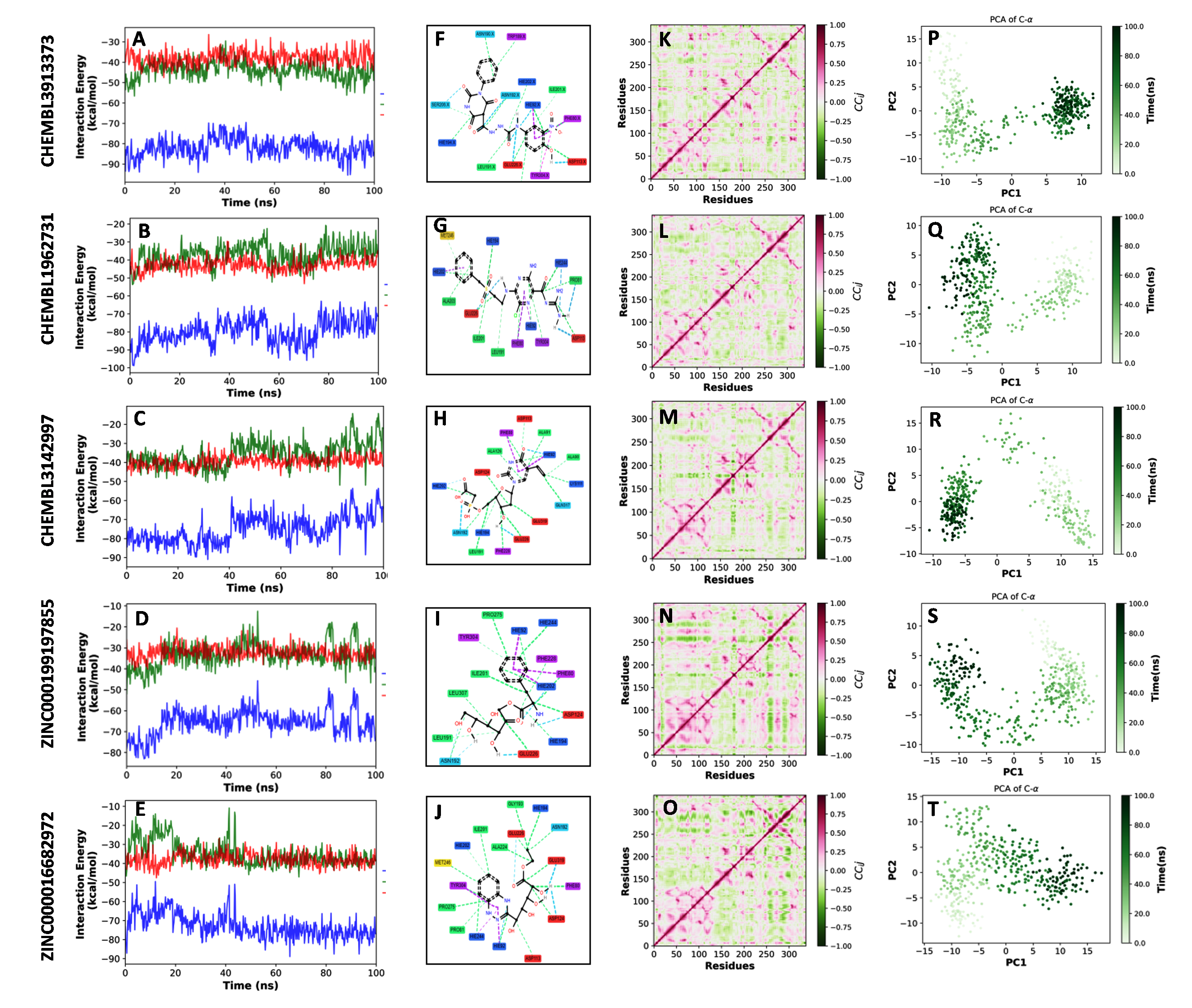

Supplement: Supplementary file 1 [file ijms-24-01412-s001.zip › Figure S10.png]

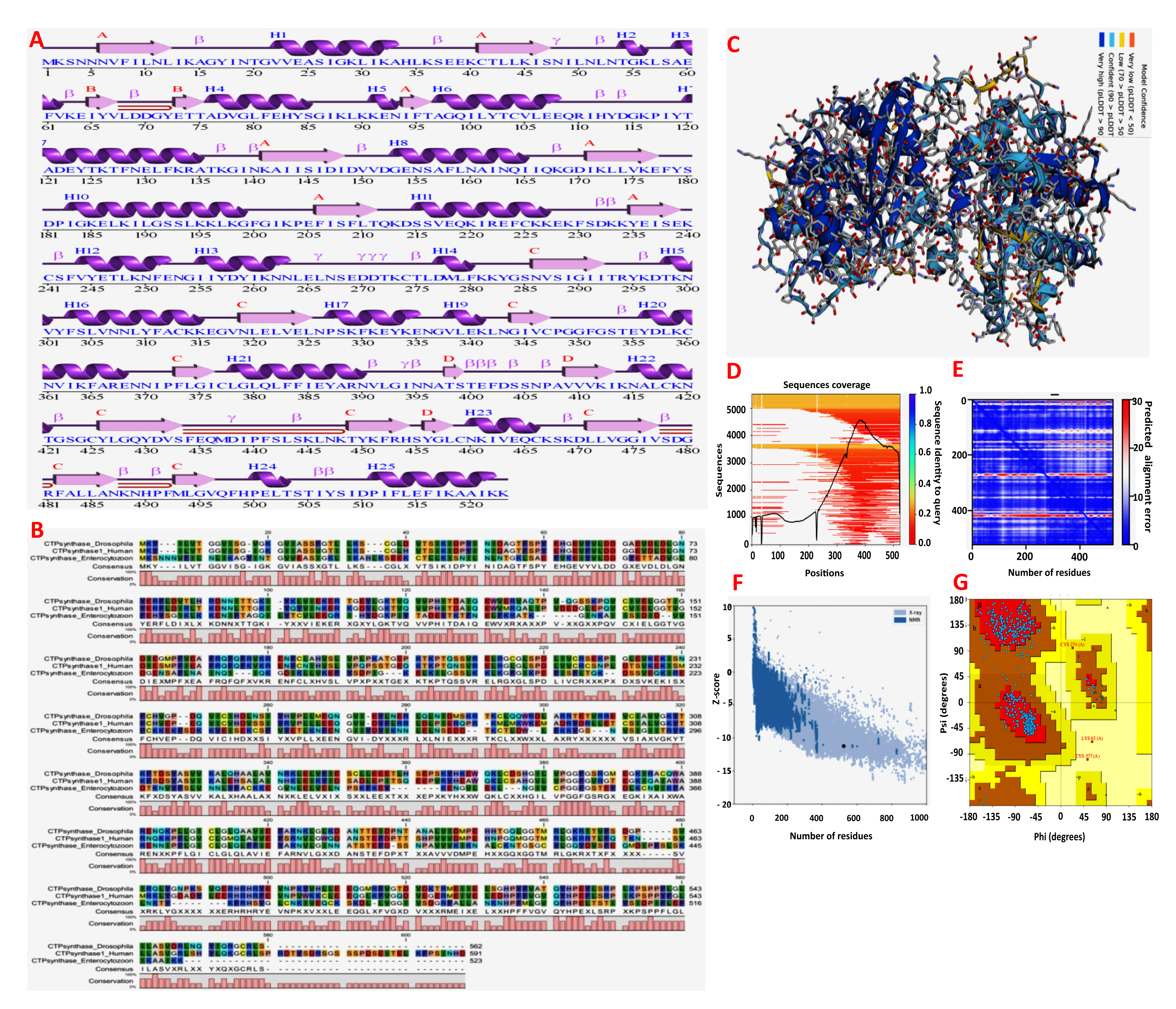

Supplement: Supplementary file 1 [file ijms-24-01412-s001.zip › Figure S2.png]

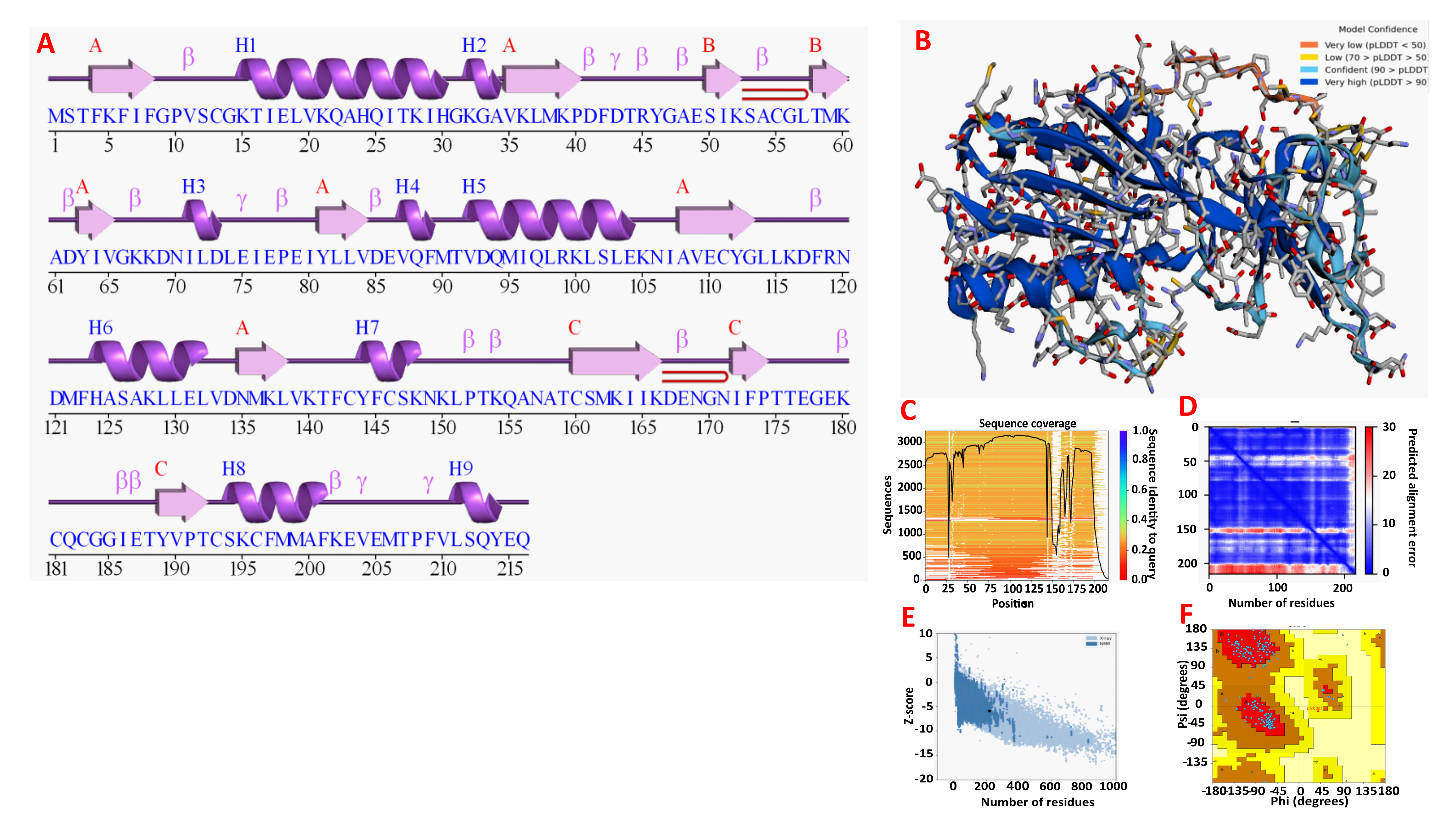

Supplement: Supplementary file 1 [file ijms-24-01412-s001.zip › Figure S3.png]

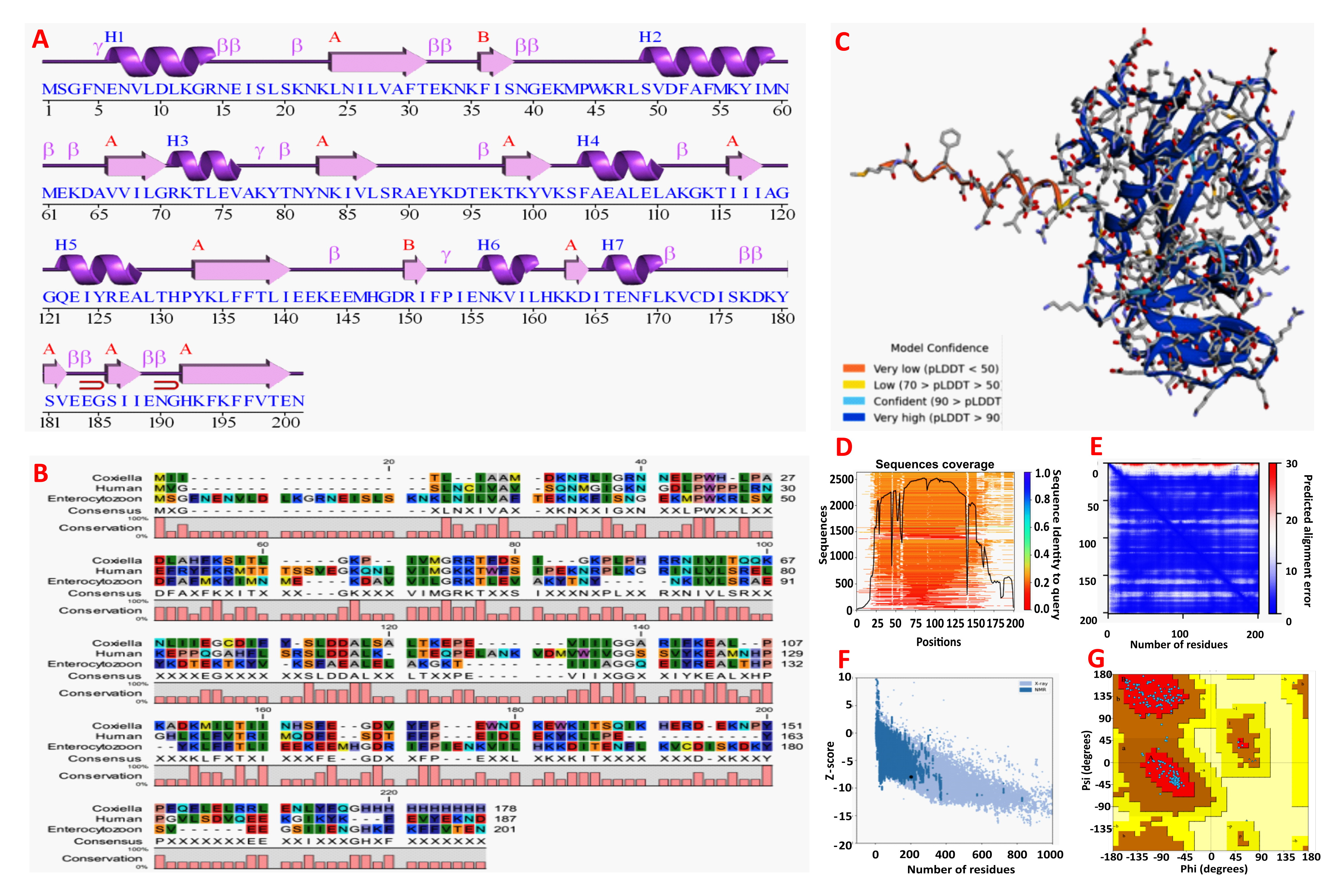

Supplement: Supplementary file 1 [file ijms-24-01412-s001.zip › Figure S4.png]

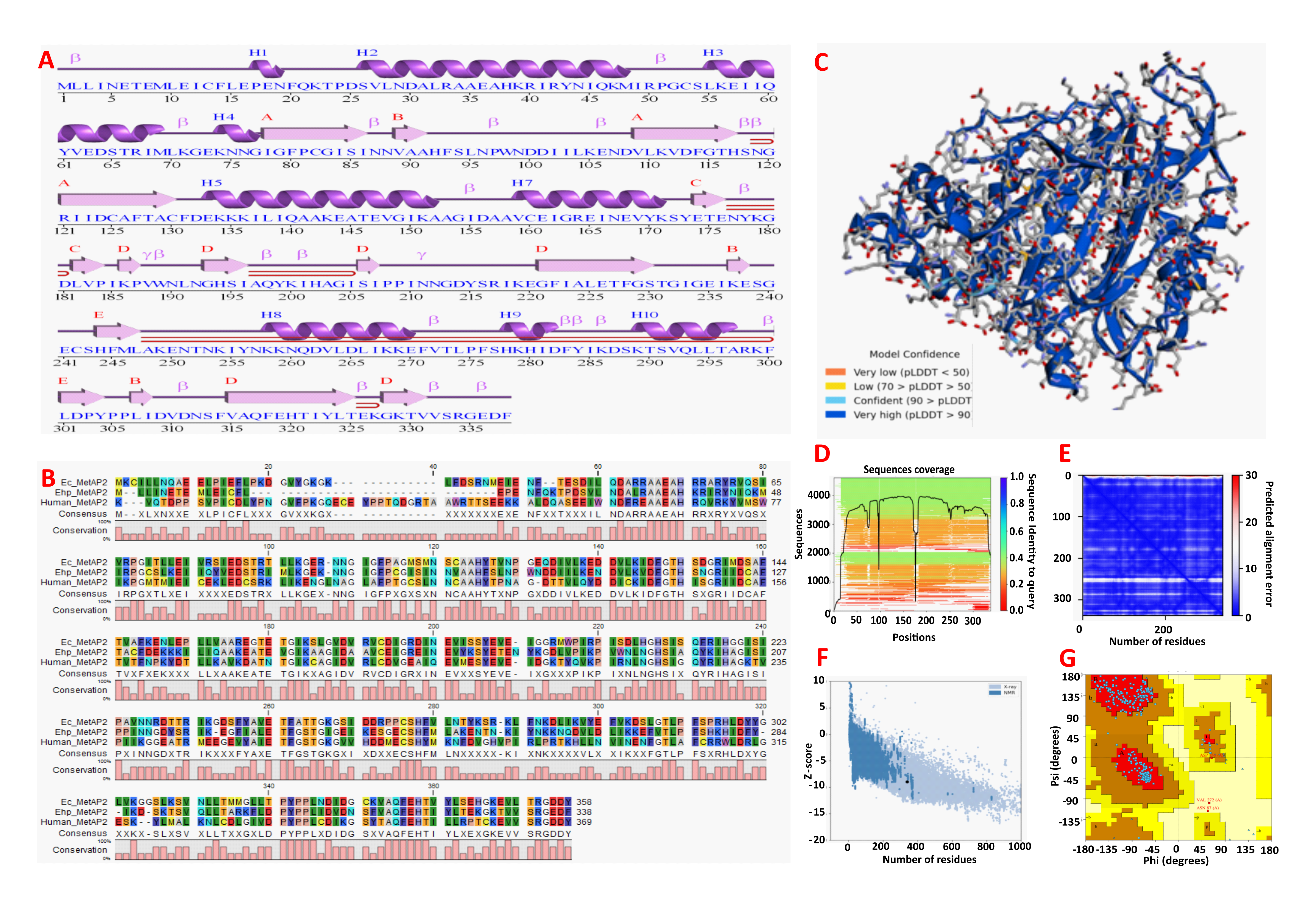

Supplement: Supplementary file 1 [file ijms-24-01412-s001.zip › Figure S5.png]

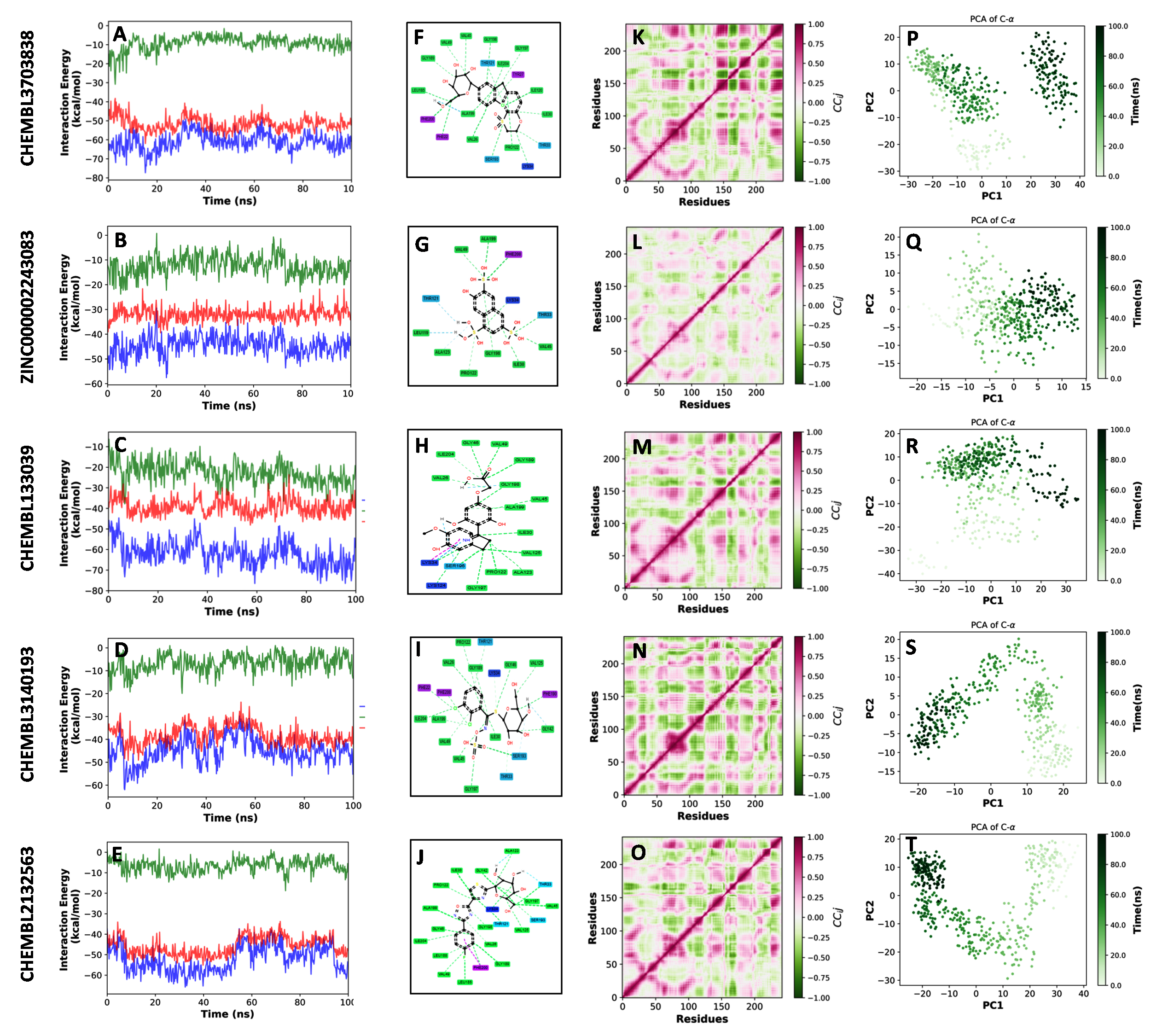

Supplement: Supplementary file 1 [file ijms-24-01412-s001.zip › Figure S6.png]

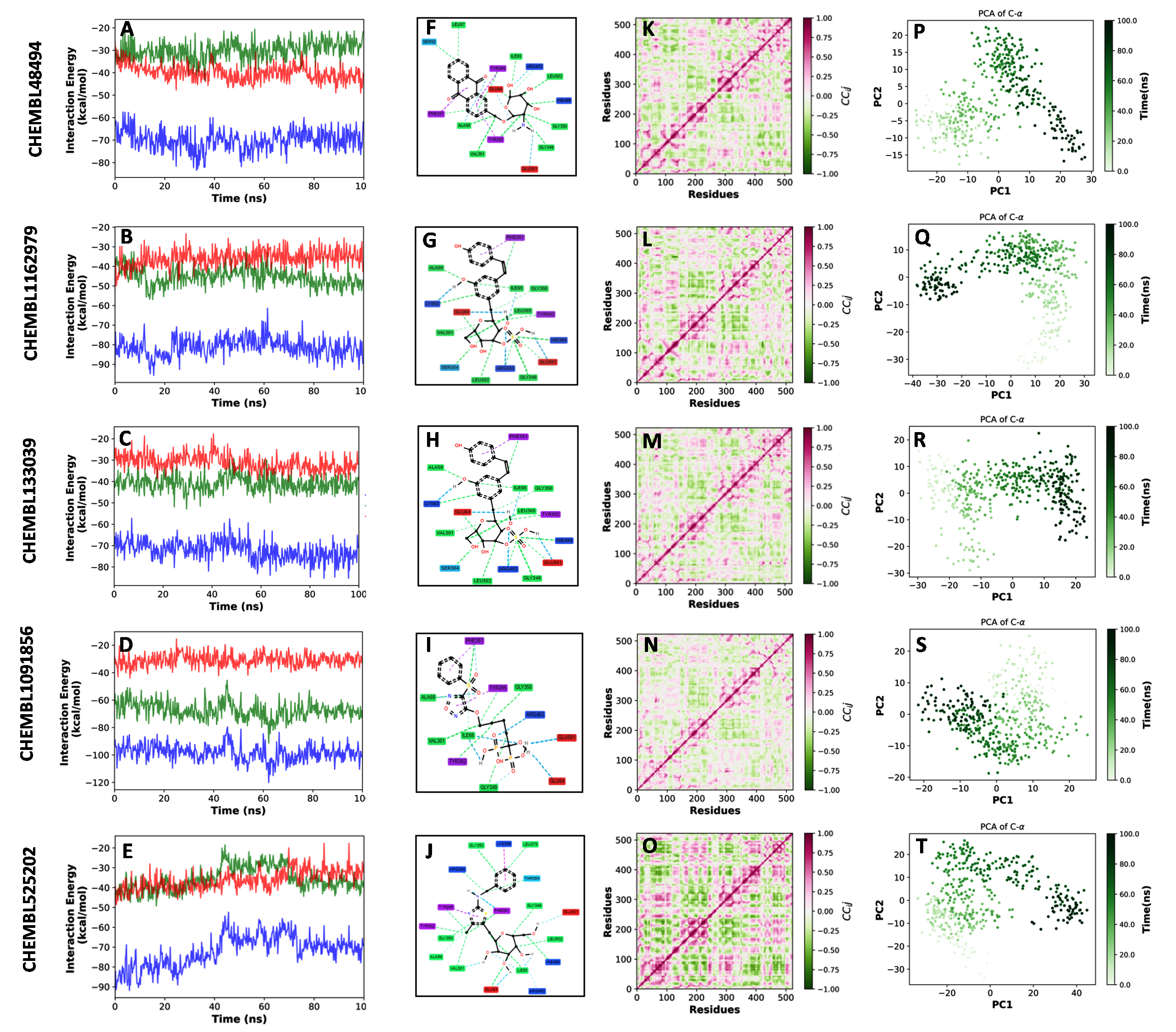

Supplement: Supplementary file 1 [file ijms-24-01412-s001.zip › Figure S7.png]

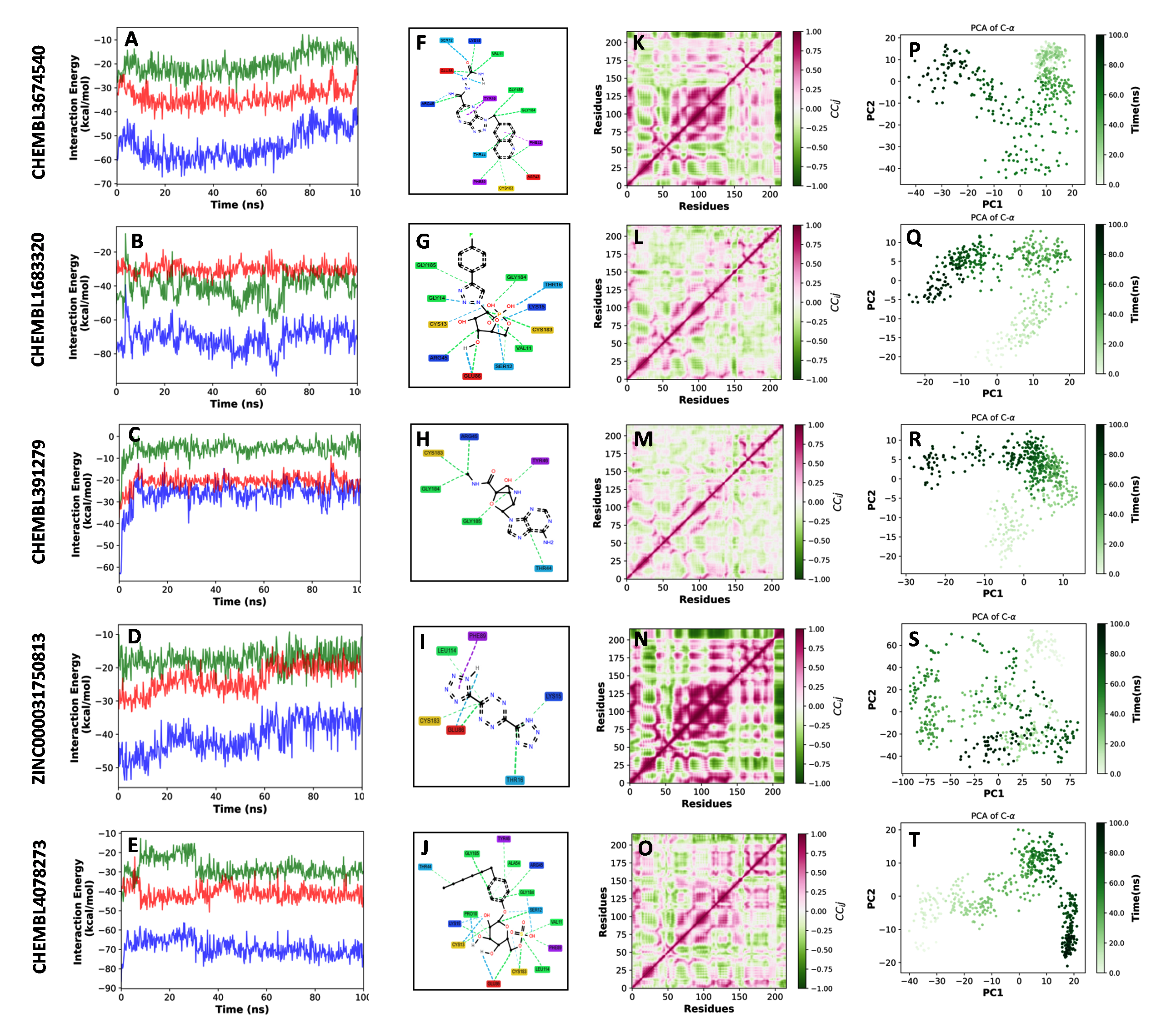

Supplement: Supplementary file 1 [file ijms-24-01412-s001.zip › Figure S8.png]

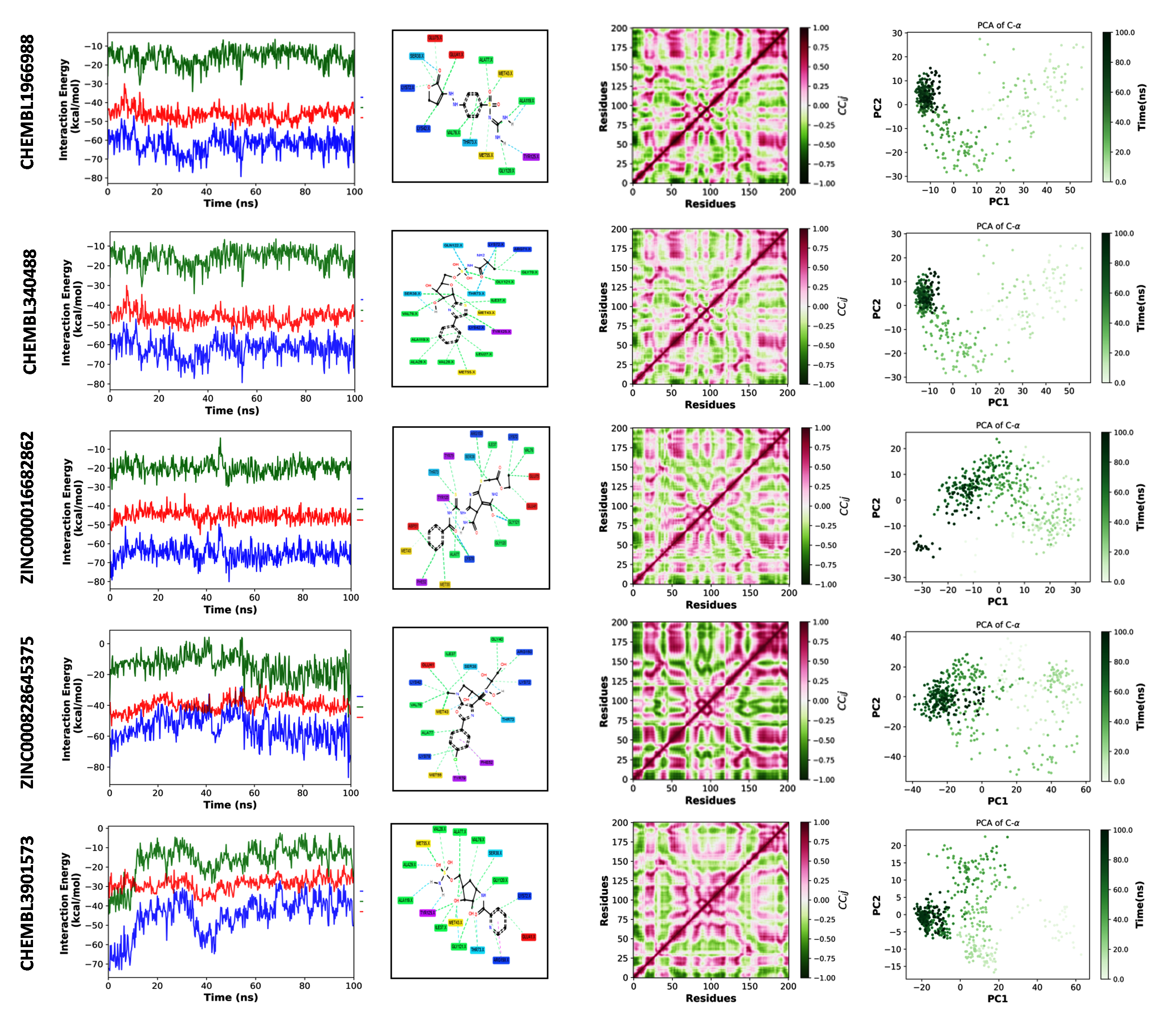

Supplement: Supplementary file 1 [file ijms-24-01412-s001.zip › Figure S9.png]
